# Irrational Use of Medicines—A Summary of Key Concepts

**DOI:** 10.3390/pharmacy4040035

**Published:** 2016-10-28

**Authors:** Richard Ofori-Asenso, Akosua Adom Agyeman

**Affiliations:** Research Unit, Health Policy Consult, P.O. Box WJ 537, Weija, Accra, Ghana; akosuaadom@gmail.com

**Keywords:** good prescribing, rational medicine use, rational dispensing

## Abstract

Medicines play an integral part of healthcare delivery. However, they are expensive commodities and account for a significant proportion of overall health expenditure in most countries. Irrational use of medicines is a major challenge facing many health systems across the world. Such practices are likely to lead to poor health delivery that may put patients at risk and result in wastage of scarce resources that could have been used to tackle other pressing health needs. The concept of “rational use of medicine” can at times be confusing and not easily appreciated by patients, healthcare providers, policy makers, or the public, all of whom need to collaborate effectively to address this challenge. In this article, we summarize basic concepts such as rational medicine use, good prescribing and dispensing, and explore some of the factors that contribute to irrational use of medicines as well as potential impacts of such practices. This article has been written with the intention of offering a clear, concise, and easy to understand explanation of basic medicine use concepts for health professionals, patients, policy makers, and the public.

## 1. Introduction

Medicines play an important role in healthcare delivery, and when used properly, can help cure diseases, relieve symptoms, and alleviate patient suffering. Nonetheless, irrational use of medicines remains a major issue facing most health systems across the world [1]. The World health organization (WHO) estimates that more than half of all medicines are inappropriately prescribed, dispensed, or sold. Additionally, around 50% of patients fail to take their medicines correctly [2]. The problem of irrational medicine use is known to be worse in developing countries with weak health systems, where mechanisms for routine monitoring of medicine use are often not well developed or are at times non-existent [3]. Promoting the rational use of medicines requires effective policies as well as efficient collaboration between health professionals, patients, and entire communities. Adequate understanding regarding the relevant aspects of medicine use on the part of all stakeholders is essential to drive collaborative efforts towards addressing the problem of irrational medicine use [4,5]. Tackling the issue of irrational medicine use is considered to be essential not only to improve healthcare delivery towards ensuring patient safety, but also to allow for optimal utilization of resources. This stems from the fact that as much as 25%–70% of overall health expenditure in developing countries is spent on medicines whereas, around 10% of health expenditure in most high-income countries is consumed by medicines [6]. In this article, we focus on summarizing basic concepts around medicine use, with the aim of providing clear and concise information for the education of health professionals, patients, policy makers, and the public.

## 2. What is Rational Use of Medicines?

The concept of the rational use of medicines is an old one, dating as far back as 300 B.C, when the Greek physician Herophilus said that “*medicines are nothing in themselves, but are the very hands of god if employed with reason and prudence*” [7]. Over the years, the rational use of medicines has come to be regarded as one of the key principles in delivering effective and quality healthcare [8,9].

In 1985, the WHO convened a meeting of experts on the rational use of drugs, out of which the rational use of medicine was defined to represent a situation where “Patients receive medications appropriate to their clinical needs, in doses that meet their own individual requirements, for an adequate period of time, and at the lowest cost to them and their community” [10]. The World Bank has also defined rational medicine use as comprising two key principles: (1) the use of drugs according to scientific data on efficacy, safety, and compliance; and (2) the cost-effective use of drugs within the constraints of a given health system [4,11].

The WHO and the World Bank definitions differ in two main areas: (1) the use of scientific data in prescribing, which appears to be more enforced in the Word bank definition; and (2) while the World Bank definition incorporates countries’ financial capacity as a consideration in medicine use, the WHO advocates for the use of medicine with the lowest cost wherever possible, irrespective of the particular health system [4].

The definitions by the WHO and the World Bank broadly assume a medical therapeutic view; however, the rational use of medicines can also be seen from the consumer or patient’s perspective. What is deemed rational from a medical perspective may be considered irrational by the patient, and vice versa [12]. It is therefore essential that both medical and consumer/patient perspectives are considered in order to gain a holistic understanding of the rational use of medicines. In this article, however, we focus mainly on the medical perspective of irrational medicine use.

**Figure 1:**
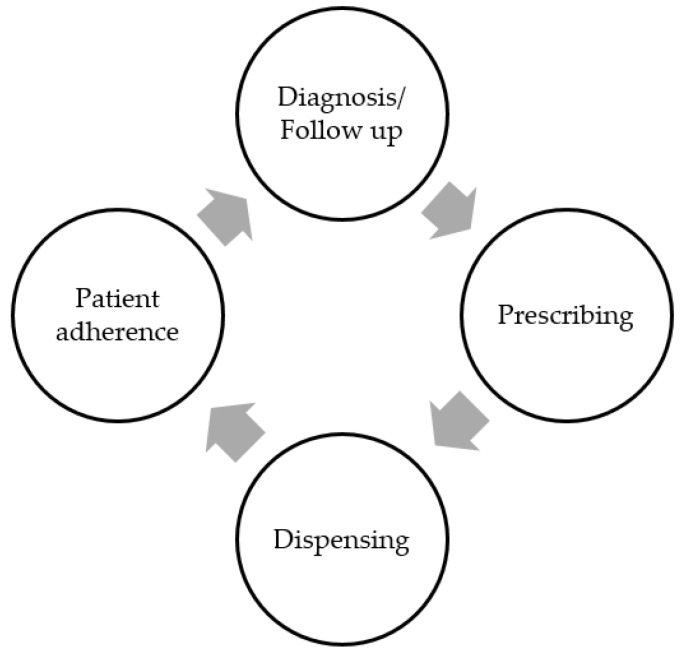
The cycle of medicine use [9].

From a medical perspective, the inappropriate use of medicines can begin at any of the four main stages of the medicines use cycle [9]. These four stages are diagnosis, prescribing, dispensing, and patient adherence (Figure 1). The diagnosis stage involves identifying and defining the problem(s) requiring intervention. This initial stage can set up a cycle of inappropriate medicine usage if the wrong problem (e.g., disease condition) is outlined for intervention. Following the establishment of a diagnosis, a treatment will usually be prescribed—this could be a pharmacological or a non-drug therapy. Subsequently, patients are supplied with the prescribed medicines, and are then expected to take the medications as directed (adherence).

There is an on-going debate as to whether the rational use of medicines should be defined as a universal concept or customized to suit individual context [4]. These discussions are borne out of the observation that countries face different challenges, and have varied health system capacities [4]. Let us use the case of the Essential Medicines List (EML) as an illustration. Essential medicines are regarded as those medicines that satisfy the priority health care needs of the population [13]. The EML is a list of minimum medicine needs for a basic health-care system, focusing on the most efficacious, safe, and cost-effective medicines for priority conditions. The EML concept is based on the notion that the use of a limited number of well-known and cost-effective medicines can lead to better health care, enhanced long-term medicines supply, and more equitable and sustainable access to products [14]. This seems to resonate very well with developing countries with scarce resources, where infrastructure is often inadequate to efficiently handle the medicine supply process (e.g., procurement, storage, and distribution) and which may require additional resources to build [4]. Within such constraints, the EML is seen as a reasonable approach to streamline medicine expenditure and free resources for infrastructure improvement [4]. However, in developed countries, there may be limited infrastructure gaps, and more resources may be available to cover medicine expenditures. As Almarsdottir and Traulsen point out, “industrialised countries can to some extent afford medicines that are new and expensive, whereas most developing countries will have to be very restrictive and keep to essential drug lists. Both these decisions can be viewed as rational in the light of each country’s economic situation” [4]. Nonetheless, the rising health care expenditure in most developed countries has reignited debates that perhaps even in these rich nations, the EML concept may still be very applicable and highly essential [15].

## 3. The Prescribing Process

Although it is frequently encountered and often perceived to be a routine activity, prescribing is a complex process—one which tests the healthcare providers’ knowledge and application of sound therapeutics principles, communication skills, as well as their approach to and appreciation of risks and uncertainties [16]. Often, the prescribing process begins with establishing the goal(s) of therapy (e.g., alleviating pain, curing an infection, or even improving appetite, etc.). Patient expectations and preferences can sometimes influence what goals are set or not set. Subsequent to the determination of goals, a treatment is then selected. Often, prescribers are confronted with the task of choosing from many options [17]. Ideally, the final pharmacological choice should be arrived at through a benefit–risk analysis based on medicine and patient factors, incorporating other issues such as availability and cost [16,18]. Patient factors that may influence the medicine selection process include physiological status (e.g., pregnancy, kidney failure) and susceptibility to adverse effects, as well as on-going drug therapy, as there may be potential for drug–drug interactions (e.g., the use of ketoconazole in patients already on atorvastatin could increase blood levels of the statin and further expose patients to high risk of liver damage and rhabdomyolysis). Drug factors that could influence selection include evidence of safety and efficacy, as well as pharmacokinetic and pharmacodynamic properties. For instance, a medicine with a once-daily dosing regimen may be preferred over one with multiple dosing for reasons of compliance [19].

The prescribing phase can at times be daunting for health professionals, especially when differences in risks and benefits of available therapies are not clear, and where guidelines are not explicit. Openness and engagement with the patient at this stage is very crucial, as invariably the patient is the ultimate recipient of any benefits or risks of taking the medication. As such, clear explanation should be offered to patients regarding the pros and cons of taking and not taking proposed medicine(s), including any uncertainties surrounding treatment [20]. For most patients, transitioning into the role of someone who has to take medicines is often a difficult process, and the presentation of a diagnosis by medical personnel only as a basis to take medicine may not be a sufficient motivator [21]. This is more so in instances where the benefits of the proposed treatment may not be immediate or unclear, such as in blood pressure control [20]. Thorough discussion with the patient on all relevant aspect of her/his condition is therefore essential to ensure that patients have confidence in the prescriber as well as the proposed therapy—without this, adherence is unlikely to be properly achieved. Perhaps the essence of this interaction between prescribers (e.g., doctors) and patients is best emphasized by Hall et al. [22] that, *“Medicine is an art whose magic and creative ability have long been recognized as residing in the interpersonal aspects of patient–physician relationship*”. Effective communication with patients is a skill that any prescriber should aspire to achieve, as this is the medium through which medical information is communicated, as well as addressing patient’s needs, expectations, and even emotions [23].

For many centuries, the medical practice has been paternalistic in nature, based on the fundamental assumption that a physician has access to information not known to the patient, and that this information will be used in a manner that generates health benefit to the patient (or perhaps do no harm) [24]. In recent decades, medical practice has transitioned from paternalism to individualism, with patient autonomy, for instance, being considered as a fundamental principle [24,25]. A number of models of the doctor–patient interaction have emerged which promote patient-centred communication and incorporate patient’s influence and preferences on medical decision-making, including for instance, the decision to prescribe [26].

## 4. What Constitutes a Good Prescribing?

While the term ”*good prescribing*” is often used in literature, its meaning remains quite elusive. This stems from the fact that, in addition to patients, there are many actors within the health sector who may conceptualize good prescribing from different perspectives [27]. A 2011 report commissioned by the King’s Fund, for instance, notes that within the UK;

“*The NHS as a whole might define it as the lowest-cost prescribing that meets public health needs. The Department of Health and commissioners are keen to monitor prescribing and may measure good prescribing according to the available information and, as this largely relates to drug costs, their definitions of good prescribing tend to use cost as the focus. The pharmaceutical industry may look on good prescribing as prescribing of the latest drug to all patients who have need of treatment on the basis that new equals better. Evidence-based practitioners tend to define it as the use of therapies proven to be most effective in randomised controlled trials (RCTs), or according to evidence-based guidelines*”.

[27]

While the above perspectives all highlight the subjectivity of what constitutes ”good prescribing”, some individuals have offered more objective definitions of the term. According to Aronson [28], a good prescribing is one that “recommends a medicine appropriate to the patient’s condition and minimizes the risk of undue harm from it”. Aronson’s definition is in accordance with Barber [29], who explained that a good prescribing is one that achieves the four aims, namely: (1) to maximize effectiveness; (2) minimize risks; (3) minimize costs; and (4) respect the patient’s choices. Barber further indicates that this conceptualization of good prescribing “brings together the traditional balancing of risks and benefits with the need to reduce costs and the right of the patient to make choices in treatment” [29].

**Figure 2:**
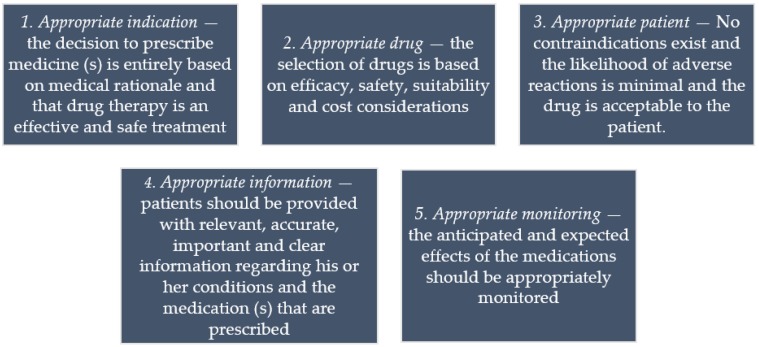
Criteria for rational prescribing promoted by the WHO [10].

Both Barber and Aronson’s conceptions agree to a large extent with the requirements for rational prescribing promoted by the WHO. The WHO has outlined five key requirements necessary for a prescribing to be regarded as good or rational (Figure 2) [10].

## 5. What is Irrational Prescribing?

Irrational prescribing refers to prescribing that fails to conform to good standards of treatment [10]. This may manifest in five different ways, namely: under-prescribing, over-prescribing, incorrect prescribing, extravagant prescribing, and multiple prescribing.

Under-prescribing indicates the instance where the medicines required are not prescribed, or an insufficient dosage or treatment duration is issued. This can occur when, for instance, an inadequate weight-based dose is administered in patients such as children [30]. In certain instances, however, doctors may after thorough consideration decide not to prescribe—this is considered rational under-prescribing [31]. In a study among Dutch general practitioners, Van den Heuvel et al. reported that in 65% of patients, prescirbing physicians after thorough consideration decided not to prescribe a specific medication. Under-prescribing can contribute to significant morbidity and mortality, although it remains an area of medicine use that has attracted less attention. Wauters et al., for instance, has reported a strong association between under-prescribing and misuse with hospitalization and death among a cohort of community-dwelling elderly people aged 80–120 years [32].

Over-prescribing refers to instances where a medicine that is not indicated is prescribed, or if indicated, the duration of treatment is too long or the quantity of medicine given to patients exceeds the amount required for the current course of therapy. This can include, for instance, giving 21 days course of an antibiotic for a minor infection that requires just 7 days of treatment, or when an antibiotic is prescribed in the first place for a suspected viral infection [33].

Incorrect prescribing also occurs when a medicine is given for the wrong diagnosis, the prescription is prepared improperly, or adjustments are not made to incorporate the patient’s co-existing medical, genetic, or environmental conditions [34]. An example is when a doctor fails to consider an allergy that a patient may have which could be triggered by a new medication being prescribed. Incorrect prescribing is also deemed to have occurred when a prescriber fails to recognize that prescribing a certain medication could react with the patient’s current therapy (e.g., prescribing serotonergic antidepressants to a patient already on a monoamine oxidase inhibitor) [35].

Extravagant prescribing is said to have occurred when a prescriber issues a more expensive medicine when a less expensive one of comparable safety and efficacy exists, or where a prescriber treats a patient symptomatically instead of tackling the underlying serious condition. An example may include writing an unnecessarily expensive cough mixture when it presents no documented extra benefits from commonly available cheaper options. Similarly, extravagant prescribing is said to have occurred when a patented product in a class is prescribed when low costs generics are available in the same class, which could have been used without compromising care. Such classes include the proton pump inhibitors (PPIs), statins, renin–angiotensin inhibitors, etc. The savings can be enormous, as demonstrated by Godman and colleagues [36,37].

Multiple prescribing is also deemed to have taken place when two or more medicines are prescribed when fewer would have achieved same effect, or where prescribers treat several related conditions when treatment of the underlying (primary) disorder would improve or cure the other conditions. For instance, prescribing for individual symptoms of Malaria when treating the underlying infection is likely to resolve the cascade of symptoms.

Although the above types of irrational prescribing occur in different frequencies across regions of the world, the WHO has outlined some commonly encountered patterns of irrational prescribing. Some of the commonly observed patterns include the excessive use of injections, multiple drug prescriptions, the excessive use of antibiotics for treating minor acute respiratory infections (mostly viral in origin), and the use of minerals and tonics for managing malnutrition [10]. This list is not exhaustive, and highlights the extent to which the inappropriate use of medicines remains a worldwide challenge [38].

## 6. The Art of Dispensing

Once prescribing is complete, the dispensing stage is when patients receive their medications. Often, the patient will present a prescription—an authority note from the prescriber instructing the recipient (dispenser) to serve the patient on what has been instructed. Dispensing is often carried out by a trained pharmacist or dispensing technicians. In clinical practice, the separation of prescribing and dispensing activities is considered to be a safety mechanism to ensure an additional independent assessment of the proposed therapy before patient begins treatment [39]. In some settings, such as rural areas with limited health personnel [40], dispensing may be carried out by the prescriber (e.g., dispensing doctors). This is considered non-ideal, and may promote irrational prescribing, especially if the prescriber stands to gain financially [3]. Wilcock found that dispensing doctors in Cornwall in England issued more prescriptions than non-dispensing doctors, and were also less likely to prescribe generic medicines [41]. Similarly, Trap et al. found that dispensing by doctors in Zimbabwe was associated with less clinically appropriate but costly prescribing [42].

In the instances where the prescribing and dispensing roles are separated, sound therapeutic knowledge on the part of the dispenser is extremely essential to cross-check any loop holes in the prescription made and make appropriate recommendations/interventions to the prescriber if necessary. The engagement between the dispenser and the patient is also key, as it can significantly impact how the medicines are used by the patients. For instance, adherence is likely to improve only if the patient understands the importance of taking the medications, can follow instructions correctly, and appreciates the risks of non-adherence.

The WHO advocates that rational dispensing principles should be followed at all times to ensure that patients receive adequate information regarding the use of dispensed medicines, so as to achieve the desired benefits [43]. For instance, if dispensing practices such as counting, packaging, and labelling are poorly executed; they are likely to impact the patient’s confidence in the dispensed products, and subsequently compliance to therapy [43]. Table 1 summarizes essential steps of dispensing to promote rational medicine use. This is by no way an exhaustive list.

## 7. Factors Contributing to the Irrational Use of Medicines

There are many factors that contribute to the irrational prescribing or use of medicines. These factors can be traced to various stages of the medicine use cycle, and can be broadly categorized into those emanating from patients, prescribers, workplace (health system), supply system (including industry influences), regulation, drug information or misinformation, or a combination of these factors [3,38]. Uninformed patients who may have the perception that there exists a pill for every ailment can exert undue pressure on health providers to prescribe medicines, even when this is not needed. The influence of patients in the prescription of certain drugs such as antibiotics has been widely documented [45]. Macfarlane et al. for instance, investigated the impact of patients’ pressure on antibiotic prescribing in the management of acute lower respiratory tract illness at 76 primary care facilities in the UK. Their results indicated that, of the patients evaluated, 74% were prescribed antibiotics, and that non-clinical factors influenced prescribing 44% of those receiving antibiotics, of which patient pressure was the reason in more than half [46]. Additionally, doctors often find it difficult to refuse prescribing for children, the elderly, persons well known to them, as well as individuals they like [47].

Regarding prescriber-related factors, irrational prescribing can arise as a result of several internal or external factors. For instance, the prescriber may lack adequate training, or there may be inadequate continuing education, resulting in the reliance on out-dated prescribing practices which may have been learnt while under training. The lack of opportunities for on-job continuing education is a challenge faced by many health professionals in resource-poor countries [3,48]. Moreover, workplace issues, such as lack of laboratory facilities typical of many resource-poor settings may promote inappropriate prescribing. For instance, a prescriber may want to conduct laboratory investigation to confirm the presence of infection, but may have to resort to empirical treatment if laboratory facilities are unavailable. Even where laboratory facilities are available, prescribers may be reluctant to use them due to other factors, such as time constraints. In a study in Ghana by Polage et al., for instance, over 90% of physicians indicated that time constraints meant that they rarely ordered tests [49]. Other issues, such as under-staffing, medicine shortages, and a lack of an inventory of a list of medicines from which choices need to be made are some of the factors known to promote irrational prescribing in many developing countries [3].

There are also practices by pharmaceutical companies that are seen to enhance irrational prescribing [50]. For instance, pharmaceutical sales representative visits to doctors have been found to not only increase the prescription of the promoted drug, but also to lead to a decrease in the market share of competitor products [51]. There is evidence to support that pharmaceutical sales representatives often exaggerate the efficacy of their products while questioning the integrity of competitor brands, and may even encourage off-label use [52]. Over-reliance on such sources of information could lead to irrational prescribing. A systematic review by Spurling et al. regarding the information from pharmaceutical companies and the quality, quantity, and cost of physicians’ prescribing identified that physician exposure to information from drug companies sometimes resulted in lower prescribing quality [50].

## 8. Impact of the Irrational Use of Medicines

The impact of irrational medicines use can vary widely. Firstly, when medicines are used inappropriately, the risks of adverse drug reactions (ADRs) is increased, especially in geriatric patients or in co-morbid individuals who may have compromised physiologic functions [53]. For instance, in a retrospective cohort study among older people in Australia, the presence of comorbidity was a strong predictor of repeat admissions for ADRs, especially in those with comorbidities which are being managed in the community [54]. The cost implications of ADRs can also be enormous [55]. In Germany, for instance [56], ADRs are estimated to cost more than €430 million annually, whereas in the UK, the cost of emergency admissions subsequent to an ADR has been estimated at £2 billion per year [57].

According to the Nobel Laureate Joshua Lederberg, “*the future of humanity and microbes will evolve as episodes...of our wits versus their genes*” [58]. Lederberg points out that bad human practice, such as the inappropriate use of antibiotics is one the key factors underlying the global insurgence of antimicrobial resistance [58]. For instance, studies have shown that subtherapeutic antibiotic concentration contributes to the development of antibiotic resistance by promoting genetic alterations, including changes in gene expression and mutagenesis [59].The occurrence of antimicrobial resistance is seen not only as a threat to the progress made in health, but one which can potentially draw humanity back to periods like the pre-antibiotic era, where many individuals suffered and died from untreatable bacterial infections [60].

**Figure 3:**
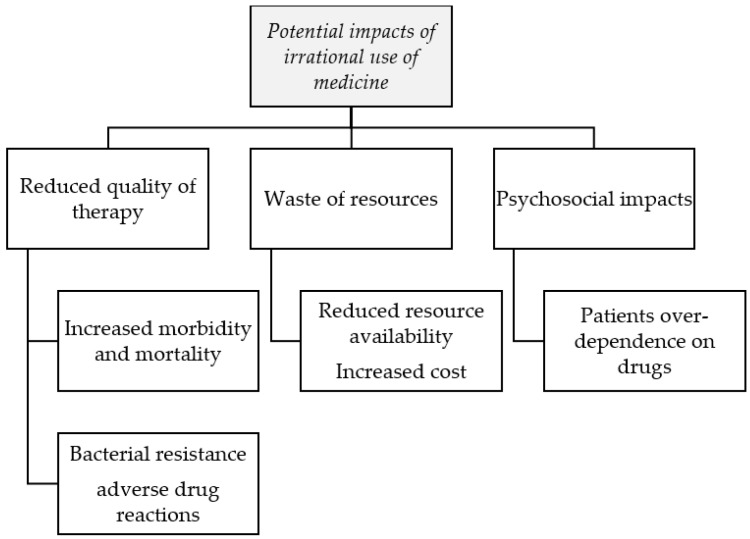
Some possible impacts of the irrational use of medicines.

Irrational prescribing can also expose patients to the possibility of developing drug dependence to certain medicines, such as pain killers and tranquillizers. Inappropriate prescribing practices such as the overuse of injections can expose patients to the contraction of certain injection-related conditions, such as abscesses, hepatitis B, and HIV/AIDS [61]. Indiscriminate prescribing of injections can also increase workload, as health professionals need to administer doses [3]. When medicines are prescribed indiscriminately, it may also exert a psychological effect on patients who may come to the conclusion that there exists “a pill for every ill”, thereby causing a cycle of excessive demand for medicines. In addition, the inappropriate use of medicines can lead to wastage of scarce health resources, which can further reduce the availability of other vital medicines or increase treatment cost. The WHO estimates that the appropriate use of medicines can result in about 50%–70% cost-efficiency in medicines expenditure [62]. The probable impacts of irrational prescribing (or use) of medicines are summarized below (Figure 3).

## 9. Strategies to Tackle Irrational Prescribing

According to the WHO, irrational prescribing is a “disease” which is difficult to treat—prevention is however possible [63].

There exist various strategies to change patients’ and prescribers’ behaviour towards the promotion of rational prescribing. These strategies can be grouped broadly as targeted or system-oriented approaches [9]. Targeted approaches comprise educational and managerial interventions, while system-oriented strategies include regulatory and economic interventions [9,64]. Educational interventions are often aimed at persuading or informing, and this usually involves the use of printed materials, seminars, or face-to-face contacts [65]. However, according to Wettermark et al. [66], educational interventions may influence prescriber knowledge and awareness, but their effectiveness in changing behaviour remains modest unless used in combination with other strategies. Managerial strategies, on the other hand, are mainly aimed at guiding practice. Such managerial interventions that may be employed include monitoring, supervision and feedback, the use of a restrictive medicines list, drug utilization reviews, or the use of structured prescription forms [64]. An example in this case is the wise list in Sweden, which is an essential medicines list (EML) with high adherence to just 200 medicines to improve physician familiarity with quality medicines and reduce costs, which is supplemented with regular physician monitoring against expert guidance [67].

Economic strategies, on the other hand, are aimed at promoting positive financial incentives while at the same time eliminating perverse incentives for prescribers [38]. Economic interventions that may be employed include the implementation of significant changes in service providers’ reimbursement schemes or disallowing prescribers to sell medicines themselves, which can remove the financial motivation for over-prescribing [9,64].

Regulatory interventions utilize laws and regulations to influence prescribers’ practices through restrictions and requirements. An example of such an approach includes allocating each medicine a minimum level of prescriber or health facility; e.g., no injectable antibiotics at primary health care centres [64], mandatory generic substitution at pharmacies [68], or requiring prior authorization before the prescription of some medicines, as is the case for the pharmaceutical benefits scheme in Australia [69].

In order for an intervention to be very effective, it must focus specifically on an identified prescribing behaviour and be targeted at the facilities or prescribers in greatest need of improvement [9,64]. In many instances, multiple interventions may have to be deployed to drive the necessary changes. Of note, again, is the fact that efforts to promote rational medicine/prescribing should be multifaceted in nature, and must also target aspects of patient and community behaviour [70,71].

## 10. Conclusions

Irrational use of medicines is a major global health challenge with significant implications for patients, healthcare systems and communities as a whole. Several factors can promote irrational use of medicine at different stages of the medicine use cycle. Understanding these factors is key to changing population behaviour, addressing prescriber and health system deficits and implementing appropriate measures. The key factors contributing to inappropriate medicines use are likely to change over time and policy makers need to be up-to-date with current trends.

## Figures and Tables

**Table 1:** Summary of relevant dispensing steps [43,44].

STEPS	ACTION
**Accept and cross-check**	*Crosscheck prescription details* Prescriber details Patient details Confirm items to be dispensed
**Review**	*Script validity/Legality* Meets legal requirement (e.g., date, signature/stamp, etc.)
	*Safety and appropriateness* Appropriate indication Safe dosing Contraindications
	*Check patients’ dispensing history* New or changed treatment Duplication Possible drug–drug interactions Compliance issues Misuse/abuse (both intended and unintended) Patient factors - Age - Allergies - Other health conditions, including pregnancy
**Prepare and check products**	*Product selection* Appropriate drug Brand Strength Formulation Quantity
	*Label and assemble dispensed products* Name of patient Generic name of dispensed drug Strength of the drug Dosage instruction in symbols or words as may be appropriate Duration of treatment Date of dispensing The name of the institution where the drug was dispensed Organize counselling aids
**Supply and counsel**	*Supply prescription to patient/carer: re-check* Correct patient? Correct medicines? Documentation present? Unusual storage/discard requirements? Patient/carer understands directions? Clarify patient/carer issues Obtain patient/carer signature for supply
